# Estimates of Toxoplasmosis Incidence Based on Healthcare Claims Data, Germany, 2011–2016

**DOI:** 10.3201/eid2708.203740

**Published:** 2021-08

**Authors:** Amrei Krings, Josephine Jacob, Frank Seeber, Uwe Pleyer, Jochen Walker, Klaus Stark, Hendrik Wilking

**Affiliations:** Robert Koch Institute, Berlin, Germany (A. Krings, F. Seeber, K. Stark, H. Wilking);; European Centre for Disease Prevention and Control, Stockholm, Sweden (A. Krings);; InGef–Institute for Applied Health Research Berlin GmbH, Berlin (J. Jacob, J. Walker);; Charité Universitätsmedizin Berlin, Berlin (U. Pleyer)

**Keywords:** Germany, toxoplasmosis, zoonoses, healthcare claims data, incidence, *Toxoplasma gondii*, parasites, food safety

## Abstract

Toxoplasmosis is a zoonotic infection contracted through *Toxoplasma gondii–*contaminated food, soil, or water. Seroprevalence in Germany is high, but estimates of disease incidence are scarce. We investigated incidences for various toxoplasmosis manifestations using anonymized healthcare claims data from Germany for 2011–2016. Patients with a toxoplasmosis diagnosis during the annual observational period were considered incident. The estimated incidence was adjusted to the general population age/sex distribution. We estimated an annual average of 8,047 toxoplasmosis patients in Germany. The average incidence of non–pregnancy-associated toxoplasmosis patients was 9.6/100,000 population. The incidence was highest in 2011, at 10.6 (95% CI 9.4–12.6)/100,000 population, and lowest in 2016, at 8.0 (95% CI 7.0–9.4)/100,000 population. The average incidence of toxoplasmosis during pregnancy was 40.3/100,000 pregnancies. We demonstrate a substantial toxoplasmosis disease burden in Germany. Public health and food safety authorities should implement toxoplasmosis-specific prevention programs.

Toxoplasmosis is caused by infection with the protozoan parasite *Toxoplasma gondii*. Transmission of *T. gondii* can occur through food items and the environment. Main infection routes are the consumption of raw or undercooked meat or meat products containing *T. gondii* tissue cysts; ingestion of *T. gondii* oocysts through contaminated food items, such as fruits and vegetables; or ingestion of oocyst-contaminated soil or water (*1*). Most (80%–90%) infections in immune-competent persons are asymptomatic or manifest with mild influenza-like symptoms (*2*). However, infections in immunocompromised persons can cause severe disease manifestations and often occur as a reactivation of latent *T. gondii* infections (*2*). Disease manifestations can include meningoencephalitis, conjunctivitis, chorioretinitis, myocarditis, pneumonitis, and hepatitis. A primary infection with *T. gondii* during pregnancy can cause severe sequelae, known as congenital toxoplasmosis, for neonates and fetuses; these manifestations may include developmental delay, blindness, epilepsy, spontaneous abortion, and stillbirth (*3*,*4*). Although *T. gondii* seroprevalence in several countries in Europe and the United States has slowly decreased over the past few decades (*5*–*8*), emerging collaborative and interdisciplinary One Health approaches may enable new prevention efforts that could substantially reduce the disease burden of toxoplasmosis.

In a systematic review, Torgerson et al. (*9*) estimated the global incidence and burden of disease for congenital toxoplasmosis as 190,100 (95% CI 179,300–206,300) cases/year, which translates into an incidence rate of 1.5 cases/1,000 live births and a burden of disease of 1.2 million disease-adjusted life years (DALYs)/year. For Europe, an incidence rate of 0.5/1,000 live births and a burden of disease of 2.8 DALYs/1,000 live births have been calculated (*9*). A meta-analysis reports a global incidence of acute toxoplasmosis during pregnancy as 1.1%, ranging from 0.5% in the European region to 2.5% in Eastern Mediterranean region (*10*).

In Germany, *T. gondii* seroprevalence was previously found to range from 20% among patients 18–29 years of age to 77% among patients 70–79 years of age (*11*). The same study estimated 345 incident congenital toxoplasmosis cases per year (*11*). In contrast, routine surveillance data in Germany from 2009–2018 identified a minimum of 6 notified cases in 2014 and maximum of 23 cases in 2008. Medical doctors are required to report congenital forms of toxoplasmosis to the Germany national surveillance system, but not other forms of the disease or its diagnosis for patients infected with HIV or receiving organ transplants. We assumed substantial underreporting of diagnosis and underascertainment, which describes the potential absence of a diagnosis (*12*). Consequently, in Germany no data are available for the incidence of toxoplasmosis manifestations other than for congenital toxoplasmosis. However, having such estimates is essential to assess the disease burden and advise on appropriate and targeted prevention measures. Toxoplasmosis testing during pregnancy is not covered by the statutory health insurance in Germany; general screening of pregnant women has been shown to be cost-effective, but self-financed screening leads to selective testing of mostly women of higher educational status (*13*,*14*). Although information about the risk for foodborne *T. gondii* infections during pregnancy is available to the public (*15*), there is no systematic monitoring of *T. gondii* in food products. 

Disease surveillance of *T. gondii* is fragmented and unreliable; valid disease estimates could consequently inform and justify implementation of preventive measures. We aimed to estimate the annual incidence of different toxoplasmosis manifestations in Germany during 2012–2016. The method for using healthcare claims data in this study followed Lykins et al.’s approach for assessing toxoplasmosis estimates in the United States (*16*).

## Methods

### Data and Definitions

We obtained the study data from the anonymized healthcare claims database and provided by the Institute for Applied Health Research Berlin (InGef). Approximately 60 of the 123 statutory health insurance providers in Germany contribute to the database, which covers longitudinal data from ≈7 million of the 83 million Germany residents. The characteristics of this dataset and its external validity have been described (*17*). The authors showed that, compared with the general population, the database population was slightly younger and includes a smaller proportion of East Germany inhabitants. However, rates of hospitalization and overall mortality and drug prescription rates were similar to those of the general population. The overall illness rates were slightly lower in the database population.

**Figure 1:**
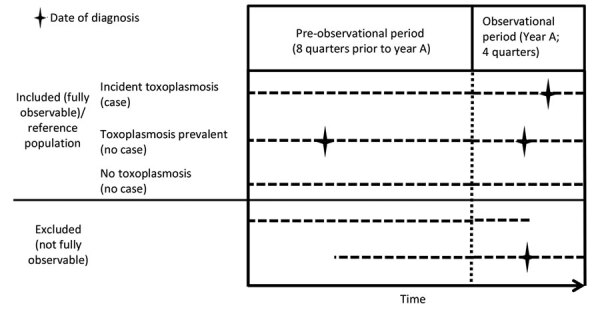
Visualization of different inclusion and exclusion definitions for study of toxoplasmosis incidence based on healthcare claims data, Germany, 2011–2016.

The study period covered 2011–2016. To estimate the annual toxoplasmosis incidence rates, we used inclusion criteria of continuous insurance with one of the statutory health insurance providers for >8 quarters before the year analyzed and for all quarters of the year analyzed or until death (Figure 1). An exception was made for toxoplasmosis in mother and child, in which children needed to be insured since birth and pregnant women for >4 quarters before and during the entire pregnancy to be included (Table 1). Case definitions for various toxoplasmosis manifestations were determined by diagnosis codes from the International Classification of Diseases, 10th Revision (ICD-10) (Table 1). The case definition for toxoplasmosis during pregnancy also includes toxoplasmosis-specific laboratory testing and therapy.

**Table 1:** Case definitions for toxoplasmosis disease manifestations used in analysis of healthcare claims, Germany*

Toxoplasmosis manifestation	Case definition
Non–pregnancy-associated	
Ocular toxoplasmosis	Patient with ICD-10 diagnosis code B58.0
Cerebral toxoplasmosis	Patient with ICD-10 diagnosis code B58.2
Hepatitis through toxoplasmosis	Patient with ICD-10 diagnosis code B58.1
Pneumonitis through toxoplasmosis	Patient with ICD-10 diagnosis code B58.3
Toxoplasmosis of other specified sites	Patient with ICD-10 diagnosis code B58.8
Unspecified toxoplasmosis	Patient with ICD-10 diagnosis code B58 or B58.9
Toxoplasmosis in mother and child	
Congenital toxoplasmosis†	Children <12 mo of age with ICD-10 code P37.1 or B58.9
Toxoplasmosis during pregnancy‡	Pregnant woman with ICD-10 code B58
	AND 1 of the following laboratory tests: avidity testing (EBM 32640) or testing for *Toxoplasma gondii* in amniotic fluid or fetal blood (EBM 32833)
	AND who received 1 of the following treatments: spiramycin (before 16th week of pregnancy), pyrimethamine in combination with sulfadiazine/clindamycin/atovaquone (after 16th week of pregnancy), trimethoprim/sulfamethoxazole

*The reference population for incidence estimation for each form of toxoplasmosis was all patients in the healthcare database not fulfilling the case definition. ICD-10, International Classification of Diseases, 10th Revision. †Reference population was children <1 year of age. ‡Reference population was female patients 15–50 y of age and pregnant. Pregnancy was defined based on ICD-10 diagnostic codes indicating birth, stillbirth, abortion, or miscarriage.

### Estimates of Incidence

Toxoplasmosis patients are considered incident if they receive a diagnosis during the observational period but not in the preobservational period. For overall estimation of case numbers and incidence in Germany, we adjusted calculations in accordance with German Federal Statistical Office estimates for age, sex, and geographic distribution for each respective year. We calculated annual incidences for each toxoplasmosis manifestation separately and the average annual incidence as the arithmetic mean of the 6 yearly-determined incidence rates for 2011–2016. Patients identified in 2016, the most recent study period, were used for stratified analysis of geographic distribution of residence, sex, and age.

### Recurring Medical Claims of Toxoplasmosis

**Figure 2:**
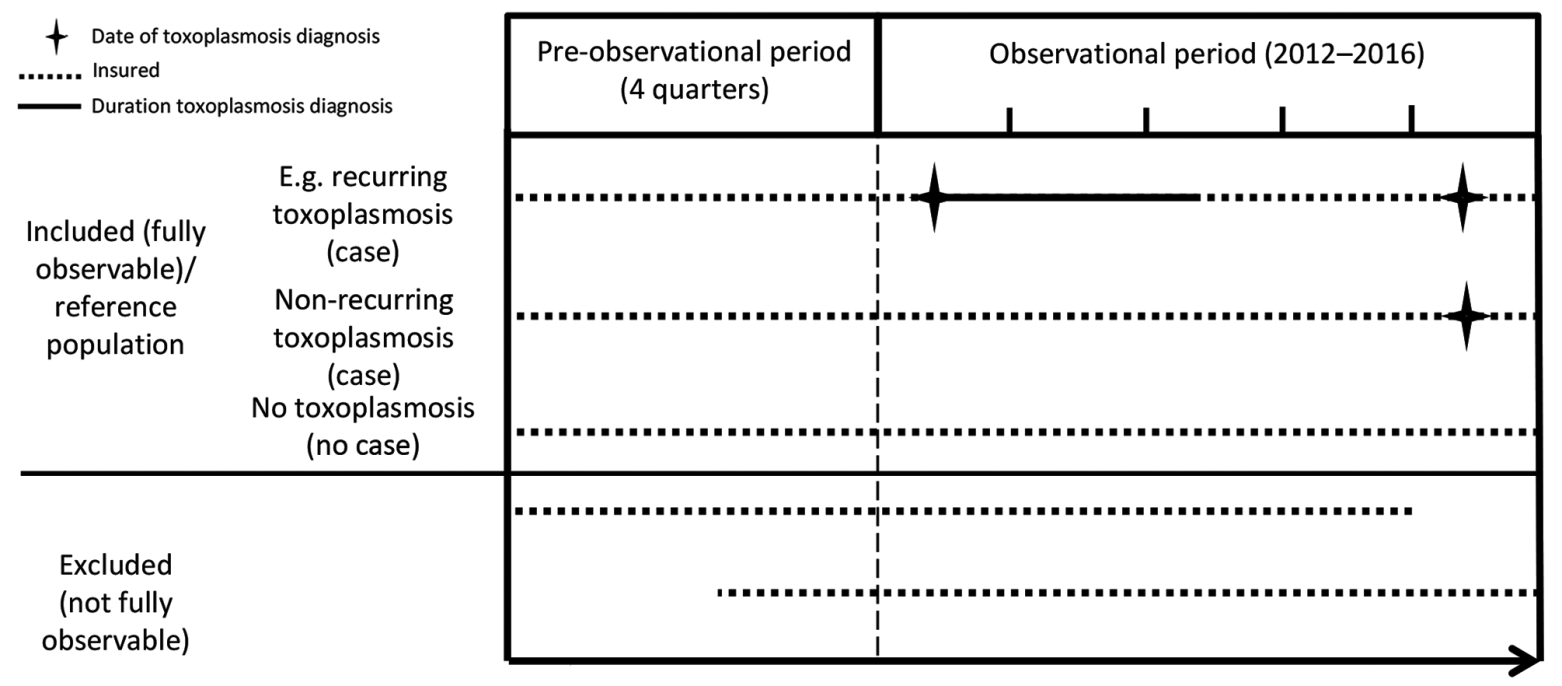
Visualization of different inclusion and exclusion definitions of recurring medical claims and identification of underlying conditions for study of toxoplasmosis incidence based on healthcare claims data, Germany, 2011–2016.

To investigate potential toxoplasmosis relapse, we used recurring medical claims as an approximation. We defined any second medical toxoplasmosis claim as recurring if the patient had >2 quarters without an existing ICD-10 code for toxoplasmosis; if the patient had a diagnosis of a different toxoplasmosis manifestation than that previously recorded; or if the patient record showed >2 quarters without treatment while they still had the ICD-10 code. We differentiated rates of recurring medical claims per 100 person-years by relapse with the same or a different toxoplasmosis manifestation. Criteria for inclusion were a fully insured 4-quarter preobservational period before the first medical claim and continuous insurance throughout the 2012–2016 study period (Figure 2). We analyzed patients with recurring medical claims as proportions of all toxoplasmosis patients.

### Underlying Conditions in Patients with *T. gondii* Infection

For comparison, we used the scientifically reported and discussed underlying conditions provided in Lykins et al. (*16*) to analyze those conditions for Germany in this analysis (Appendix Table 1). We defined toxoplasmosis cases as described previously (*16*) (Table 1). Toxoplasmosis-negatives were cases insured without any recorded episode of toxoplasmosis. Inclusion criteria were similar to those for the assessment of recurring medical claims (Figure 2); we excluded pregnant women and children <12 months of age from this subanalysis. We matched the toxoplasmosis and reference groups 1:1 on the basis of age (by 5-year age groups), sex, and quarter of the diagnosis date of the underlying illness. Matching by quarter of diagnosis helped to avoid confounding due to seasonal variation of some underlying conditions. We calculated odds ratios for measure of association based on the frequencies of predefined conditions among the toxoplasmosis and reference group. 

## Results

### Study Population 

Of ≈7 million persons insured during 2011–2016, we determined that 4.8–5.2 million were eligible for inclusion in our analysis per year. We found 2,625 toxoplasmosis patients who met the case definitions in the database for these years, which is equivalent to 48,368 patients among ≈83 million Germany residents. This total corresponds to an average annual case number of 8,061 patients. For 950/2,625 (36%) of patients, a classification in one of the specific toxoplasmosis manifestations was possible; most patients had no specified toxoplasmosis manifestation.

### Temporal and Geographic Distribution

**Figure 3:**
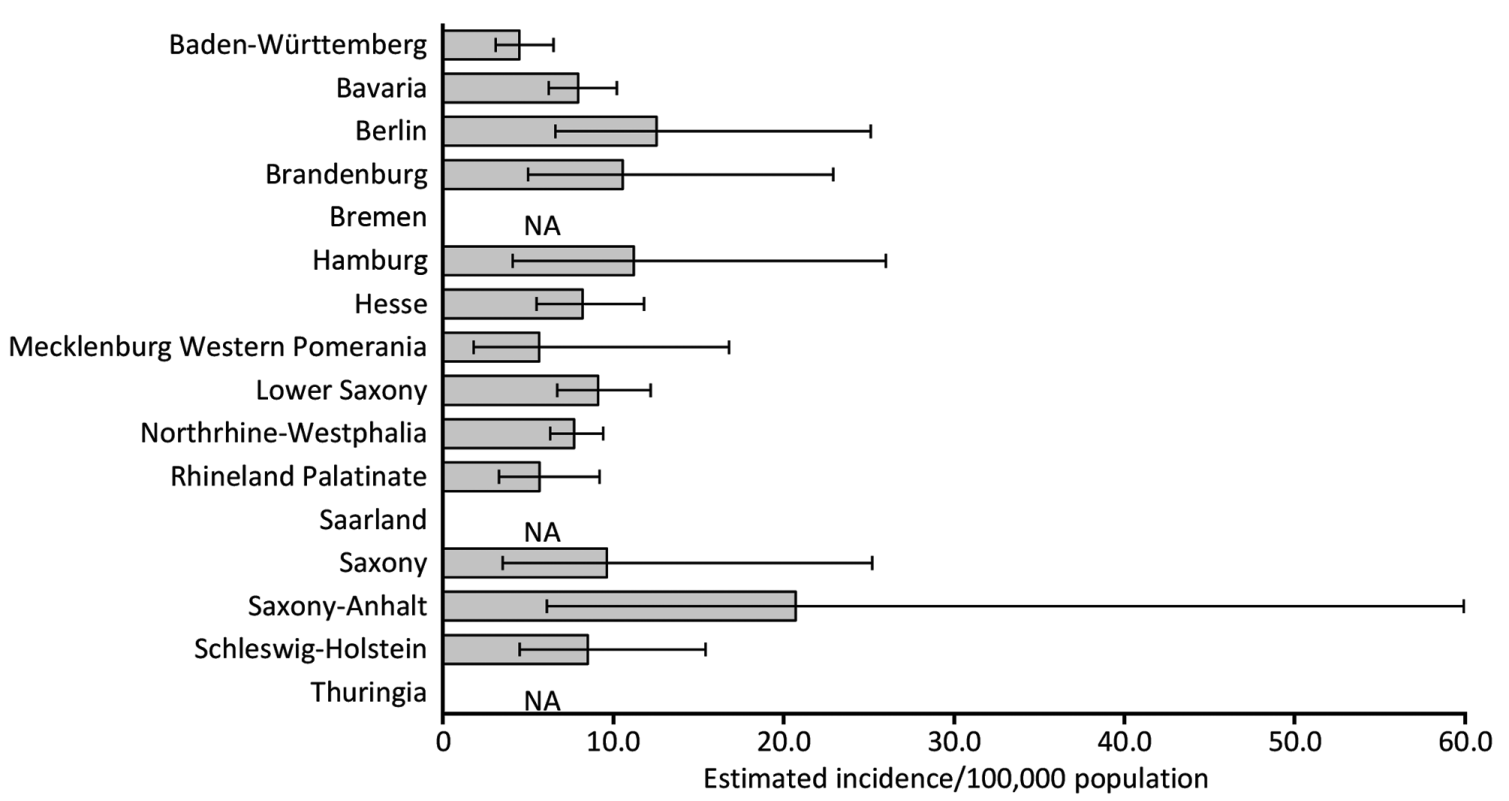
Toxoplasmosis disease incidence by federal state in Germany, 2016. Error bars indicate 95% CIs. No estimates were available for Bremen, Saarland, and Thüringen. NA, not available.

Incidence for non–pregnancy-associated toxoplasmosis was as low as 8.0 (95% CI 7.0–9.4)/100,000 population in 2016 and as high as 10.6 (95% CI 9.4–12.6)/100,000 population in 2011. The average annual incidence of non–pregnancy-associated toxoplasmosis cases was 9.5/100,000 population. Geographically, in 2016, the incidence of 4.5 (95% CI 3.1–6.5)/100,000 population in Baden-Württemberg was significantly lower than the incidence of 12.5 (95% CI 6.6–25.1)/100,000 population in Berlin and 9.1 (95% CI: 6.7–12.2)/100,000 population in Lower Saxony (Figure 3). 

### Ocular Toxoplasmosis

We estimated an average annual case number of 1,601 cases of ocular toxoplasmosis and an average annual incidence of 2.0/100,000 population in Germany. The estimated incidence of ocular toxoplasmosis fluctuated between 1.5 (95% CI 1.1– 2.3) patients/100,000 population in 2016 and 2.5 (95% CI 2.0–3.8) patients/100,000 population in 2013 (Table 2). In 2016, the highest incidences of ocular toxoplasmosis were seen among women and in the 51–60-year age group (Table 3).

**Table 2:** Estimated incidence of toxoplasmosis manifestations by year, Germany, 2011–2016

Disease manifestation	Year	No. patients in database	No. patients identified	Estimated no. patients (95% CI)	Estimated cases/100,000 population (95% CI)
Ocular toxoplasmosis	2011	4,705,497	86	1,618 (1,253–2,940)	2.0 (1.6–3.7)
	2012	4,751,579	95	1,937 (1,522–3,189)	2.4 (1.9–4.0)
	2013	5,024,715	111	2,017 (1,623–3,029)	2.5 (2.0–3.8)
	2014	5,134,795	74	1,359 (1,015–2,241)	1.7 (1.3–2.8)
	2015	5,177,282	76	1,452 (1,035–2,342)	1.8 (1.3–2.9)
	2016	5,171,212	75	1,224 (941–1,931)	1.5 (1.1–2.3)
Cerebral toxoplasmosis	2011	4,705,497	8	144 (56–1,534)	0.2 (0.1–1.9)
	2012	4,751,579	6	114 (40–1,377)	0.1 (0.1–1.7)
	2013	5,024,715	5	97 (24–1,090)	0.1 (0.0–1.4)
	2014	5,134,795	6	80 (32–934)	0.1 (0.0–1.2)
	2015	5,177,282	13	218 (115–970)	0.3 (0.1–1.2)
	2016	5,171,212	10	200 (74–883)	0.2 (0.1–1.1)
Other types of toxoplasmosis†	2011	4,705,497	45	865 (NA)	NA
	2012	4,751,579	40	715 (NA)	NA
	2013	5,024,715	32	588 (NA)	NA
	2014	5,134,795	58	912 (NA)	NA
	2015	5,177,282	44	782 (NA)	NA
	2016	5,171,212	43	651 (NA)	NA
Nonspecified types of toxoplasmosis	2011	4,705,497	306	5,866 (5,093–7,406)	7.3 (6.3–9.2)
	2012	4,751,579	283	5,503 (4,703–6,981)	6.8 (5.8–8.7)
	2013	5,024,715	265	5,177 (4,483–6,389)	6.4 (5.6–7.9)
	2014	5,134,795	237	4,277 (3,686–5,327)	5.3 (4.5–6.6)
	2015	5,177,282	309	5,821 (5,070–6,936)	7.1 (6.2–8.4)
	2016	5,171,212	242	4,569 (3,862–5,628)	5.5 (4.7–6.8)
Congenital toxoplasmosis	2011	97,177	10	74 (32–1,478)	0.1 (0.0–1.8)
	2012	98,140	5	43 (8–1,321)	0.1 (0.0–1.6)
	2013	100,420	10	73 (32–1,066)	0.1 (0.0–1.3)
	2014	103,481	7	77 (16–934)	0.1 (0.0–1.2)
	2015	105,882	11	116 (41–879)	0.1 (0.1–1.1)
	2016	107,517	6	65 (25–751)	0.1 (0.0–0.9)
Toxoplasmosis during pregnancy	2011	63,102	18	252 (144–485)	29.3 (16.7–56.4)
	2012	53,178	14	226 (110–478)	32.0 (15.6–67.7)
	2013	53,476	16	324 (171–619)	44.4 (23.4–84.9)
	2014	52,951	21	296 (176–560)	40.5 (24.1–76.6)
	2015	53,900	21	450 (247–803)	60.3 (33.0–107.5)
	2016	37,302	14	186 (99–431)	35.3 (18.7–81.7)

*NA, not available. †Other types include hepatitis, pneumonitis, other organs. Because the number of cases is summarized, no 95% CI and incidence estimation are available.

**Table 3:** Estimated incidence of ocular toxoplasmosis manifestations by age group, sex, and region, Germany, 2016*

Characteristic	No. patients in database	No. patients in country (95% CI)	No. cases/100,000 population (95% CI)
Sex			
M	34	516 (346–948)	1.3 (0.9–2.3)
F	41	707 (494–1,389)	1.7 (1.2–3.3)
Age group			
<1 y	<5	NA	NA
1–5 y	<5	NA	NA
6–10 y	<5	NA	NA
11–20 y	5	70 (22–329)	0.9 (0.3–4.1)
21–30 y	9	160 (73–432)	1.6 (0.7–4.3)
31–40 y	9	157 (65–421)	1.5 (0.6–4.1)
41–50 y	11	178 (81–430)	1.6 (0.7–3.7)
51–60 y	22	354 (212–653)	2.7 (1.6–5.1)
61–70 y	8	131 (56–399)	1.4 (0.6–4.2)
>71 y	5	94 (28–779)	0.8 (0.2–6.4)
Region			
East	6	172 (58–861)	1.1 (0.4–5.3)
West	69	1,052 (810–1,400)	1.6 (1.2–2.1)
North	20	415 (244–819)	2.0 (1.2–3.9)
Middle	33	492 (331–1,157)	1.7 (1.2–4.1)
South	22	317 (197–559)	1.1 (0.7–1.9)
Total	75	1,224 (941–1,931)	1.5 (1.1–2.3)

*Reference population N = 5,171,212. NA, not available.

### Cerebral Toxoplasmosis

The average annual number of cerebral toxoplasmosis patients in Germany was 142, and the average annual incidence was 0.18/100,000 population. Incidence for cerebral toxoplasmosis was 0.1 cases/100,000 population in 2012, 2013, and 2014 and 0.3 cases/100,000 population in 2015 (Table 2). Because very few cases were recorded in the database, we were unable to stratify estimates for most sociodemographic characteristics (Table 4).

**Table 4:** Estimated incidence of cerebral toxoplasmosis manifestations by age group, sex, and region, Germany, 2016*

Characteristic	No. patients in database	No. patients in country (95% CI)	No. cases/100,000 population (95% CI)
Sex			
M	5	108 (24–533)	0.3 (0.1–1.3)
F	5	92 (25–778)	0.2 (0.1–1.9)
Age group, y			
<1 y	0	0	0
1–5 y	0	0	0
6–10 y	0	0	0
11–20 y	0	0	0
21–30 y	0	0	0
31–40 y	0	0	0
41–50 y	5	123 (29–395)	1.1 (0.3–3.4)
51–60 y	<5	NA	NA
61–70 y	<5	NA	NA
>71 y	<5	NA	NA
Region			
East	<5	NA	NA
West	8	113 (46–319)	0.2 (0.1–0.5)
North	<5	NA	NA
Middle	<5	NA	NA
South	<5	NA	NA
Total	10	200 (74–883)	0.2 (0.1–1.1)

*Reference population N = 5,171,212. NA, not available.

### Other Types and Nonspecified Types of Toxoplasmosis

The average annual number of patients with toxoplasmosis manifestations at other sites, including those with hepatitis or pneumonitis from toxoplasmosis, was 752 patients. The average annual number of unspecified toxoplasmosis patients was 5,202 and the average annual incidence was 6.4/100,000 population. In 2016, toxoplasmosis incidence was significantly higher among patients who were 21–40 years of age compared with patients <21 years or >40 years of age (p<0.05) (Table 5).

**Table 5:** Estimated incidence of other unspecified toxoplasmosis manifestations by age group, sex, and region, Germany, 2016*

Characteristic	No. patients in database	No. patients in country (95% CI)	No. cases/100,000 population (95% CI)
Sex			
M	64	1,181 (863–1,730)	2.9 (2.1–4.3)
F	221	4,039 (3,384–5,048)	9.7 (8.1–12.1)
Age group, y			
<1 y	0	0	0
1–5 y	0	0	0
6–10 y	<5	NA	NA
11–20 y	18	430 (226–812)	5.4 (2.8–10.1)
21–30 y	80	1,704 (1,306–2,247)	16.9 (12.9–22.2)
31–40 y	83	1,399 (1,083–1,846)	13.8 (10.7–18.2)
41–50 y	46	582 (422–865)	5.1 (3.7–7.5)
51–60 y	33	540 (356–873)	4.2 (2.8–6.8)
61–70 y	9	149 (68–419)	1.6 (0.7–4.5)
>71 y	14	390 (102–1,235)	3.2 (0.8–10.1)
Region			
East	35	1,466 (932–2,430)	9.1 (5.8–15.0)
West	250	3,754 (3,292–4,314)	5.7 (5.0–6.5)
North	73	1,564 (1,203–2,116)	7.5 (5.7–10.1)
Middle	109	1,896 (1,413–2,773)	6.7 (5.0–9.7)
South	98	1,423 (1,152–1,795)	4.9 (4.0–6.2)
Total	242	4,569 (3,862–5,628)	5.5 (4.7–6.8)

*Reference population N = 5,171,212. NA, not available.

### Congenital Toxoplasmosis

The average annual number of congenital toxoplasmosis patients was 8.2 and average annual incidence was 0.1/100,000 population in Germany. Incidence estimates ranged from 0.05 (95% CI 0.01–1.64)/100,000 pregnancies in 2012 to 0.14 (95% CI 0.05–1.07)/100,000 in 2015 (Table 2). Our ability to estimate stratified incidence was limited because the number of cases found was low (Appendix Table 2).

### Toxoplasmosis during Pregnancy

The average annual number of patients with toxoplasmosis during pregnancy is 289 and average annual incidence is 40.3/100,000 pregnancies in Germany. Incidence of toxoplasmosis during pregnancy fluctuated between 29.3 (95% CI 16.7–56.4)/100,000 pregnancies in 2011 and 60.3 (95% CI 33.0–107.5)/100,000 pregnancies in 2015 (Table 2).

Stratification by age group and geographic region was partly limited because of low case numbers. Therefore, estimation was only possible for women 31–40 years of age, who had an estimated incidence rate of 52.7 (95% CI 24.1–138.1)/100,000 pregnancies. Stratification by region fluctuated between 34.4 (95% CI 17.7–71.9)/100,000 pregnancies in the western region of Germany and 58.2 (95% CI 17.2–177.9)/100,000 pregnancies in the northern region. No estimations are available for the middle and eastern regions (Table 6).

**Table 6:** Estimated incidence of pregnancy-associated toxoplasmosis by age group and region for 2016, Germany

Characteristic	No. patients identified in database	No. patients in country (95% CI)	No. cases/100,000 pregnancies (95% CI)
Age group, y			
15–20 y	<5	NA	NA
21–30 y	<5	NA	NA
31–40 y	10	134 (61–351)	52.7 (24.1–138.1)
41–50 y	<5	NA	NA
Region			
East	<5	NA	NA
West	12	145 (75–303)	34.4 (17.7–71.9)
North	5	78 (23–238)	58.2 (17.2–177.9)
Middle	<5	NA	NA
South	6	73 (27–176)	37.3 (13.7–89.8)
Total	14	186 (99–431)	35.3 (18.7–81.7)

*Reference population N = 37,302 women 15–50 years of age. NA, not available.

### Recurring Medical Claims of Toxoplasmosis

Among all toxoplasmosis patients found in the dataset (n = 2,776), 722 (26%) had a recurring toxoplasmosis medical claim. The highest proportion of these (250/574; 44%) was seen among patients who initially received a diagnosis of ocular toxoplasmosis. The rate of recurring medical claims (termed rate of claims in this study) among initial ocular toxoplasmosis patients was 19.8/100 person-years for any type of toxoplasmosis. Among these patients, the rate of claims in patients with an additional episode of ocular toxoplasmosis was 16.8/100 person-years; for other manifestations, the rate of claims was 3.0/100 person-years.

### Underlying Conditions

We calculated odds ratios for statistically significant underlying conditions found among toxoplasmosis cases, compared with matched controls based on 5-year age groups and sex (Table 7). Conditions that were significantly associated with toxoplasmosis were anxiety, epilepsy, lymphadenopathy, and thrombocytopenia, as well as vision loss or blindness (all p<0.05). The conditions with <5 cases in the reference group but >10 cases in the toxoplasmosis group were HIV/AIDS; memory loss; and encephalitis, myelitis, or encephalomyelitis. We tested for other conditions that were not significantly associated with toxoplasmosis or affected <5 persons in either group (Appendix Table 1). 

**Table 7:** Underlying conditions among patients with ocular, cerebral, or other toxoplasmosis manifestations, Germany, 2012–2016

Condition	Odds ratio (95% CI)
Epilepsy	2.1 (1.3–3.3)
Visual loss, blindness, etc.	2.7 (1.7–4.3)
Anxiety	1.5 (1.2–1.8)
Thrombocytopenia	3.2 (1.7–6.1)
Lymphadenopathy	6.0 (3.9–9.3)

## Discussion

Our study of toxoplasmosis incidence estimates and its manifestations, as determined from healthcare claims data, adds a valuable contribution to the evidence base. Most of the evidence on *T. gondii* infections and disease in Germany available to date is drawn either from serosurveys or from mandatory disease surveillance for congenital toxoplasmosis (*11*,*12*). Therefore, our estimation of ≈8,000 annual toxoplasmosis patients among 83 million residents of Germany offers a new assessment.

Our analysis indicates a potentially declining incidence of non–pregnancy-associated toxoplasmosis as well as toxoplasmosis during pregnancy in 2011–2016, except in 2015. This finding is in line with decreasing infections in the Netherlands (*5*) and France (*6*), as well as decreasing seroprevalence observed in Switzerland (*7*) and the United States (*8*). We hypothesize that, in France and the Netherlands, decreasing incidence is a result of improved practices in meat production, modern farming systems, increased use of frozen meat by consumers, or changes in food habits (*5*,*6*). Changing diets may also play a role in the decreasing incidence in our study; vegetarianism was shown to be negatively associated with *T. gondii* seropositivity in Germany (*11*). The observed nonsignificant increase of disease incidence in 2015 might be explained by a fluctuation of *T. gondii* exposure because, during this time, no programs were introduced by public health or veterinary health services that may have affected the number of diagnoses. We are not aware of potential changes from the healthcare sector regarding medical claims policies. Disease incidence in some Germany federal states seems higher, despite lacking statistical significance. Geographic differences in raw meat consumption in Germany, such as consumption of the regional specialty food Hackepeter, were previously shown in a cross-sectional survey (*18*). These observations are in line with the geographic differences of toxoplasmosis incidence found in our analysis.

For congenital toxoplasmosis the mandatory disease surveillance system reported 6–20 cases of congenital toxoplasmosis each year, 2011–2016 (*12*). Our analysis found 43–116 cases in Germany for the same years, confirming the suspicion that the surveillance system likely has underascertainment and underreporting. Our analysis, limited to children <12 months of age, might still underestimate the incidence of congenital toxoplasmosis compared with the postulated annual total of 345 neonates in Germany, because of potential development of clinical symptoms later in life (*11*,*19*,*20*). Another reason for an underestimation could be the lack of systematic screening of infants for congenital toxoplasmosis, which could prevent difficulties with diagnosis (*21*). Nevertheless, our analysis provides an improved estimation compared with the mandatory disease surveillance system.

Among non–pregnancy-associated toxoplasmosis, most patients were not further specified by disease manifestation, resulting in a large proportion of underascertainment; this phenomenon was similarly observed by Lykins et al. (*16*). Ocular toxoplasmosis was the non–pregnancy-associated disease manifestation with the highest incidence seen in our analysis, which is also in line with the results for the United States (*16*). The annual incidence in Germany of 2.0/100.000 population is roughly double the incidence reported by Lykins et al. in the United States. We would expect an even higher incidence in Germany, given the ≈4 times higher seroprevalence in Germany (49.1%) compared with the United States (12.4%) (*11*,*22*). The large number of patients in our dataset with unspecified toxoplasmosis probably contributes to this remaining underestimation compared to seroprevalence in Germany.

Our data on disease relapse also reveal a high relapse for ocular toxoplasmosis. These results are not surprising because ocular toxoplasmosis is common in immune-competent patients (*24*,*25*), constituting most of the population. It can develop during childhood or adolescence even if infected neonates were born without symptoms for congenital toxoplasmosis (*19*,*20*,*26*). An estimated ≈2% of *T. gondii* infections lead to ocular toxoplasmosis (*23*); this would translate into 22 patients/100,000 population annually according to the seroprevalence seen in Germany in 2008 (*11*). We see a lower average annual incidence of 2 patients/100,000 population in this analysis, possibly resulting from underdiagnosis and missing specification of toxoplasmosis cases. We further suspect an underestimation of incidence for cerebral as well as other types of toxoplasmosis, including pneumonitis and hepatitis or other organs affected, especially in immunocompromised patients; these are opportunistic infections among this group of patients and may therefore remain unrecognized.

Analysis of underlying conditions shows an association with psychiatric conditions (anxiety) and alternative diagnoses (visual loss, lymphadenopathy). Our analysis found an association with the known conditions HIV/AIDS and encephalitis, myelitis, or encephalomyelitis. However, we were unable to provide odds ratios for these conditions because of the low number of patients with HIV or encephalitis in our reference population. Directionality of conditions remains unclear from this analysis, and we were not able to confirm most associations found by Lykins et al. (*16*) on the basis of our dataset.

One limitation of this study is that healthcare claims are primarily intended for financial reimbursement rather than disease surveillance or clinical research. Therefore, the results rely on accurate coding and diagnosis and should be interpreted cautiously. Comparisons between the approaches in the United States and our data are hampered by the fundamental differences between the health systems, particularly in relation to medical claims. For similar reasons, an analysis based on toxoplasmosis-specific treatment as conducted by Lykins et al. was not possible due to different clinical guidelines. An estimate of annual toxoplasmosis incidence during pregnancy from this study (40.3/100,000 pregnancies) was restricted to women receiving toxoplasmosis-specific treatment. We can assume an unknown number of additional women need diagnostic clarification because of toxoplasmosis infection suspicion, which is likely to substantially burden the health system but is not accounted for in our incidence estimation. Although we tried to eliminate falsely diagnosed toxoplasmosis relapses by applying the criteria on patients with recurring medical claims, we need to interpret these results with caution, bearing in mind the nature of healthcare claims data and the purpose of claims. Although the estimates are adjusted for age, sex, and regional distribution, the selection of health insurance providers and the socioeconomic status of their target populations represented in this dataset may have a residual effect on these estimates that we cannot account for. Furthermore, as shown by Andersohn et al. (*17*), the overall death rate in the database population was slightly lower than the general population in Germany. This difference may also lead to an underestimation of toxoplasmosis illness rates in our analysis.

The incidence estimates for the different toxoplasmosis manifestations in this analysis provide a clearer picture of this disease’s occurrence in Germany. The average annual number of ≈8,000 toxoplasmosis patients can be regarded as high, and even more undiagnosed cases are likely. Using healthcare claims data may also help other countries with improved assessments of their toxoplasmosis burden and renew the discussion for prevention measures in European Union countries and beyond. The incidence shown and the severity of symptoms and long-term sequelae of infections justify this urgent need.

Because *T. gondii* is a parasite with transmission and disease aspects affecting the veterinary, human, and environmental medicine sectors, an overall prevention program needs to target different levels, following an international One Health approach (*27*). Screening of pregnant women is one possible method of prevention, but its cost-effectiveness, health consequences for mother and child, and effectiveness of resulting treatment are debated in light of decreasing disease incidence (*28*,*29*). Although informing pregnant women of food- and animal-related risks is important, as is currently done in Germany, the incidence found in our analysis raises doubts about the effectiveness of this method. So far, insufficient evidence for effectiveness of educational efforts targeted at pregnant women has been published (*30*). Therefore, screening should be evaluated on the basis of the national health system structure and incidence in the country in question. Other preventive strategies currently debated include screening and implementing of biosafety precautions for animal farms, as well as decontaminating meat products used for raw or undercooked consumption (*31*). A social cost–benefit analysis in the Netherlands has shown that freezing meat products is effective to reduce disease (*32*); freezing could be further implemented, especially for the production of meat products that are typically consumed raw in Germany.

Appendix Additional information about estimating toxoplasmosis incidence based on healthcare claims data, Germany, 2011–2016.
