# Supplementary material for: Estimates of Toxoplasmosis Incidence Based on Healthcare Claims Data, Germany, 2011–2016
Source: Emerg Infect Dis. 2021 Aug;27(8):2097–106. doi: 10.3201/eid2708.203740 (PMC8314822; doi:10.3201/eid2708.203740)
Supplement: Appendix — Additional information about estimating toxoplasmosis incidence based on healthcare claims data, Germany, 2011–2016. [file 20-3740-Techapp-s1.pdf]

Article DOI: https://doi.org/10.3201/eid2708.203740

Estimates of Toxoplasmosis Incidence Based on Healthcare Claims Data, Germany, 2011–2016

Appendix

Appendix Table 1. Underlying conditions reported by Lykins et al.*

Condition	Odds ratio (95% CI)†
Communicating hydrocephalus	–
Obstructive hydrocephalus	–
Idiopathic normal pressure hydrocephalus	–
Congenital hydrocephalus	–
Epilepsy	2.1 (1.3–3.3)
Bipolar disorder	–
Schizophrenia	0.9 (0.4–2.1)
Premature labor	0.7 (0.4–1.2)
Fetal growth retardation	2.2 (0.8–6.3)
Alzheimer's disease	1.3 (0.5–3.8)
HIV disease	–
Visual loss, blindness, etc	2.7 (1.7–4.3)
Multiple sclerosis	0.6 (0.2–1.4)
Benign Brain neoplasm	–
Malignant brain neoplasm	–
Anxiety	1.5 (1.2–1.8)
Substance abuse	1.2 (0.9–1.5)
Thrombocytopenia	3.2 (1.7–6.1)
Septicemia of the neonates	–
Amyloidosis	–
Memory loss	–
Impulsive disorder	–
Lupus erythematosus	–
Systemic lupus erythematosus	–
Lymphadenopathy	6.0 (3.9–9.3)
Regional enteritis (Crohn's disease)	–
Encephalitis, myelitis, encephalomyelitis	–

*Lykins J, Wang K, Wheeler K, Clouser F, Dixon A, El Bissati K, et al. Understanding toxoplasmosis in the United States through "large data" analyses. Clin Infect Dis. 2016;63:468–75. PubMed https://doi.org/10.1093/cid/ciw356
†Odds ratios are calculated for conditions occurring in $\geq$5 patients of the toxoplasmosis population and the reference population.

Appendix Table 2: Estimated incidence of congenital toxoplasmosis by sex and region for 2016, Germany*

Characteristic	No. patients identified in database	No. congenital patients (95% CI)	No. congenital disease patients /100,000 pregnancies (95% CI)
Sex			
M	5	55 (16–190)	13.6 (3.9–47.2)
F	<5	NA	NA
Age <1 y	6	65 (22–206)	8.3 (2.8–26.1)
Region			
East	<5	NA	NA
West	5	48 (14–140)	7.5 (2.3–21.9)
North	<5	NA	NA
Middle	<5	NA	NA
South	0	0	0
Total	6	65 (25–751)	0.1 (0.0–0.9)

*Reference population = 107,517 persons. NA, not available.
